# Delusional Disorder in Old Age: A Hypothesis-Driven Review of Recent Work Focusing on Epidemiology, Clinical Aspects, and Outcomes

**DOI:** 10.3390/ijerph19137911

**Published:** 2022-06-28

**Authors:** Alexandre González-Rodríguez, Mary V. Seeman, Eduard Izquierdo, Mentxu Natividad, Armand Guàrdia, Eloïsa Román, José A. Monreal

**Affiliations:** 1 Department of Mental Health, Mutua Terrassa University Hospital, 5 Dr Robert Square, 08221 Terrassa, Spain; eizquierdo@mutuaterrassa.cat (E.I.); mnatividad@mutuaterrassa.cat (M.N.); aguardia@mutuaterrassa.cat (A.G.); eloisaroman@mutuaterrassa.es (E.R.); jamonreal@mutuaterrassa.cat (J.A.M.); 2 University of Barcelona, CIBERSAM, 08221 Terrassa, Spain; 3 Department of Psychiatry, University of Toronto, 605 260 Heath Street West, Toronto, ON M5T 1R8, Canada; mary.seeman@utoronto.ca; 4 Institut de Neurociències, UAB, 08221 Terrassa, Spain

**Keywords:** delusional disorder, psychosis, antipsychotics, treatment, elderly

## Abstract

The theme, strength, and duration of a delusion are considered important in distinguishing one psychosis of old age from another. Research results, however, are mostly based on studies conducted on one form of psychosis, namely schizophrenia. The aim of this hypothesis-driven narrative review is to gather clinically important information about the psychosis identified as delusional disorder (DD), as it affects persons of senior age. We hypothesized that DD becomes relatively prevalent in old age, especially in women; and that it is associated with demonstrable brain changes, which, in turn, are associated with cognitive defects and poor pharmacological response, thus increasing the risk of aggression and suicide. Computerized searches in PubMed and ClinicalTrials.gov were conducted using the following search terms: (delusional disorder) AND (elderly OR old OR aged OR psychogeriatrics). A total of 16 recent studies (including case reports) were reviewed. Our hypotheses could not be definitively confirmed because research evidence is lacking. In order to improve eventual outcomes, our literature search demonstrates the need for more targeted, well-designed studies.

## 1. Introduction

Delusional disorder (DD) is a severe mental illness that, in order to fulfil current diagnostic criteria, requires the presence of monothematic delusions that last at least one month [1]. Affective symptoms and cognitive difficulties are traditionally not part of the presentation, although recent work points to impairments in both of these domains [2].

DD has been conventionally subtyped according to delusional themes [1]. Kendler [3] reviewed the historical concept and clinical features of paranoia that have evolved since DSM-III, the third edition of the Diagnostic and Statistical Manual of Mental Disorders, to their current status as DD in DSM-5. Currently, systematized delusions, nonprominent hallucinations and marked ideas of references are considered to best characterize DD, while a chronic course and a lack of functional deterioration are supportive of the diagnosis. No distinction is made in the literature between middle-age onset versus onset in late age [3].

The American Psychiatric Association (APA) [4] puts the lifetime prevalence of DD at around 0.02%, which is low compared to other psychotic conditions such as schizophrenia or affective psychosis. However, evidence for epidemiological data is sparse and dependent on the nature of the study population and on the choice of diagnostic criteria used [3]. A classic epidemiological paper [5] suggests that prevalence in the elderly is double that of the 2013 APA rate. This conclusion was based on results of the MRC-ALPHA study, a cohort study of an age- and gender-stratified sample of community residents 65 years of age, which used DSM-III-TR criteria for DD. The prevalence rate was found to be 0.04% and the yearly incidence of new and relapsed cases was approximately 16 per 100,000 population [5].

Several studies, such as the Halle Delusional Syndromes (HADES) study [6] have contrasted DD with other nonorganic psychoses. Results showed a prevalence of depressive symptoms of 57.1% in women and 54.5% in men with DD, underscoring the relevance of affective symptoms in this condition. More recently, de Portugal and collaborators [7] did a symptom-factor analysis in 86 individuals suffering from DSM-IV DD. Four separate psychopathological dimensions were identified: paranoid, cognitive, affective and schizoid. The cognitive dimension was associated with nonprominent hallucinatory phenomena, poor global functioning and comorbid somatic disease, especially evident with increased age. The affective dimension was associated with somatic delusions, the presence of comorbid depressive or anxiety disorders and a risk of suicide. This is consistent with a recent study by Muñoz-Negro et al. [8] of patients with schizophrenia (SZ), DD and schizoaffective disorders, in which psychopathological dimensions were determined by exploratory and confirmatory analysis. Five dimensions emerged from this model, and they explained 57% of the variance. The same research group investigated neurocognitive performance in patients with DD in comparison to healthy controls [9] and found deficits in executive and memory functions even in relatively young patients with DD.

Clinical correlates of dimensions of delusional experience have been examined in patients with SZ and compared with those of DD [10]. Patients with SZ presented relatively higher levels of disorganization and bizarreness in their delusions but lower levels of belief conviction. The delusions were less likely to spread into new areas, and the felt pressure of the delusional experience was less strong. The impact of age was not investigated.

As human beings are living longer and longer, research in psychogeriatrics has increased and has shown that biological, sociocultural, psychological and environmental factors intersect to produce mental illness in late age [11]. In particular, SZ spectrum disorders in old age are now better studied, nosologically and clinically, and treatment guidelines are available [12,13]. DD is included in the SZ spectrum, but may have characteristics of its own, which have received comparatively little attention.

Old age is acknowledged as a period of vulnerability for a first occurrence of psychosis and also for the exacerbation of previous psychotic symptoms [14]. Nomenclature and age cutoffs remain fluid but the International Late-Onset Schizophrenia Group [12] achieved a consensus that has proved influential: that late-onset SZ refers to illness onset after age 40, and very-late-onset schizophrenia refers to onset after age 60. Recent work has reviewed and compared the main clinical characteristics of early-onset, late-onset SZ, DD and mood disorders [15]. However, despite the growing interest in old-age psychosis, very few studies have specifically focused on DD.

### Aim

The aim of this literature review is to confirm or disconfirm the following hypotheses about DD in old age:

(1) That incidence and prevalence and severity of symptoms increase with age;
(2) That in old age, women are more vulnerable to DD than men;
(3) That brain changes and cognitive defects predict poor treatment response;
(4) That DD in old age is associated with self-harm and harm to others.

## 2. Materials and Methods

### 2.1. Search Strategy

A.G.-R. and M.V.S. conducted a computerized search on PubMed and ClinicalTrials.gov databases from 2012 to February 2022 in English, Spanish, French, and German. The following search terms were used: (delusional disorder) AND (elderly OR old OR aged OR psychogeriatrics). Additionally, reference lists of initially identified papers were manually checked to locate particularly relevant studies, even when published prior to 2012. In addition, for comparative purposes, we looked at reviews of studies of SZ in older age. The last search was conducted on the 4 March 2022.

### 2.2. Inclusion Criteria

Eligibility criteria for study inclusion: (1) Inclusion of participants with DD who fulfilled DSM or ICD criteria, (2) DD patients 60 years old or older, (3) publication in a peer-reviewed journal, (4) in English, Spanish, French or German, and (5) inclusion of data on epidemiology, risk factors, clinical features, imaging results or treatment outcomes, irrespective of the study design. Patients diagnosed with an organically based disorder were excluded from analysis. Those for whom the diagnosis was definitively late-onset SZ were also excluded.

### 2.3. Data Collection and Extraction

**Figure 1:**
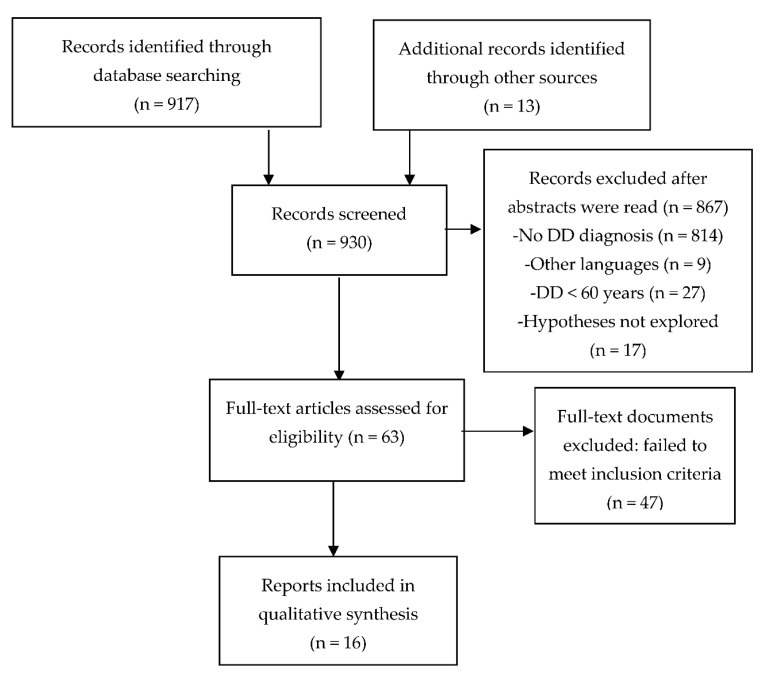
Flow diagram of included studies.

The electronic search strategy, data collection and extraction were performed independently by A.G.-R. and M.V.S. The remaining authors participated in the review of reference lists of initially included studies to identify other relevant papers. Disagreements were resolved by consensus. The screening and selection processes are illustrated in Figure 1.

### 2.4. Data Synthesis

Data were grouped according to their ability to address our original hypotheses. We then carried out a qualitative synthesis of the included studies, as summarized in Table 1. Tentative conclusions on epidemiological findings, risk factors, clinical and imaging features and treatment outcomes are detailed in Table 2.

## 3. Results

A total of 930 publications were first identified via abstracts on PubMed (n = 917) and other sources (n = 13). Sixty-three full-length studies were retained for further screening. Of these, 16 studies fulfilled eligibility criteria.

The quality of the included studies was low to moderate, as there were no clinical trials or meta-analyses specifically focused on DD.

Of the included studies, one was observational, four were register-based longitudinal studies, five were cross-sectional studies and a final six were case reports (Table 1).

### 3.1. Prevalence and Sex Ratio in Elderly DD (Hypothesis 1 and 2)

We found three studies investigating the prevalence, incidence and sex ratio in elderly patients with DD.

A register study by the Brazilian Unified Health System Information Technology Department (DATASUS) attempted to determine mental health epidemiology in elderly Brazilians by studying rates of outpatient mental health visits [16]. Data were derived from 1,124,244 outpatient visits of persons over age 60 in five regions of Brazil between 2008 and 2012. Files were grouped by diagnostic blocks. Rates of visits per 100,000 inhabitants (according to a 2010 census) were calculated for each region and were highest in the Southeast and Northeast regions. “Delusional disorders” (which include SZ and related disorders) came second (after mood disorders) as reasons for mental health visits, but the authors conceded that the data could not establish true community prevalence. This is because disorders such as SZ and delusional disorders require regular visits to outpatient services, which inflates their presence in the analyses. These results suggest, but do not prove, that the prevalence of DD is higher than expected in persons over 60 years of age. There was no mention of sex-related prevalence.

González-Rodríguez et al. [17] carried out a prospective observational study with a 24-month follow-up. The participants were 80 women diagnosed with DD as per DSM-IV-TR criteria. Psychopathological symptoms were assessed at baseline and after 6 and 24 months with the Positive and Negative Syndrome Scale (PANSS) for psychotic symptoms, the 17-item Hamilton Rating Scale for Depression (HRSD-17) for depressive symptoms and the Columbia Suicide Severity Rating Scale (C-SSRS) for suicidality. The sample was divided into two groups: premenopausal onset (n = 25) and postmenopausal onset (n = 55). A total of 57 women were available for the 24-month follow up. In women with premenopausal onset, erotomanic themes predominated; somatic and jealousy delusions were more common postmenopausal-onset women. No statistically significant differences were found with regard to negative symptoms between the two groups and current age at the time of study inclusion. Premenopausal-onset women showed higher depression scores. Postmenopausal onset appears to be more frequent than premenopausal onset, which could be due to hormones or to age.

Meesters et al. [18] carried out a register study of a psychiatric catchment area in Amsterdam to determine one-year prevalence of SZ, schizoaffective disorder and DD in patients 60 years and older. The study used DSM-IV-TR; 297.1 criteria ascertained by the use of the Mini-International Neuropsychiatric Interview Plus. Ages of onset and male/female sex ratio were also examined. From a total sample of 183, 8 patients were diagnosed with DD. The estimated one-year prevalence for DD was 0.03%, and DD was found only in women aged 40 or older at the onset of disease. The number of DD patients was, unfortunately, very small.

In summary, the prevalence of DD in old age appears to be underestimated in community or service-based studies. Although sex ratios have also been underreported, most cases in women appear to begin after menopause.

### 3.2. Relationship of Brain Structure, Cognition and Treatment Response (Hypothesis 3)

Eleven studies were found on brain structure and cognitive impairment in elderly patients with DD, some bearing on treatment response.

Nagendra and Snowdon [19] conducted a study over a 12-year period of medical records of patients with DD over age 65 referred to an old-age psychiatry service in Australia. The investigators were interested in antipsychotic use and treatment outcome. Fifty patients who fulfilled DSM-IV-TR and ICD-10 criteria for DD were recruited. Participants with a suspected diagnosis of SZ, substance-use disorder or cognitive disorder were excluded. Persecutory delusions were the most common (n = 48; 87.3%), followed by delusions of jealousy (n = 6; 11%), then somatic delusions (n = 1; 1.8%). Nonprominent hallucinations were reported in 18%, and depressive symptoms in 22%. Social isolation was significantly positively associated with DD. Of the 44 patients who were followed up (mean duration 36.6. months), 27 received antipsychotic medication. A total of 20% of the sample achieved recovery (defined as the resolution of symptoms) and 35% showed clinically significant improvement (reduction in delusion-induced distress). The majority of those who improved (96%) received antipsychotics. At follow up, four patients had developed dementia and two were diagnosed with mild cognitive impairment, but it was not clear whether or not cognitive outcomes were related to treatment response.

Krämer et al. [20] carried out a brain-imaging study in 14 patients with DD somatic-type, 18 patients with nonsomatic DD, 18 patients with schizophrenia (SZ) and 32 healthy controls. Cerebellum-optimized segmentation images were obtained by MRI. The group of DD somatic-type participants was composed of six men and eight women whose mean age was 72.6 (SD = 9.3 years). Delusional parasitosis was the main delusional theme. Comorbid medical conditions were noted in seven individuals. None fulfilled diagnostic criteria for SZ. All received antipsychotic treatment (mean chlorpromazine equivalents = 188.7, SD = 101.0) The nonsomatic DD group included 5 males and 13 females with a mean age of 55.9 (SD = 15.3). In this group, three were unmedicated. The HC group consisted of 15 males and 17 females with a mean age of 57.4 (SD = 12.4) years. Between-group comparisons were adjusted for age, gender, chlorpromazine equivalents and illness duration. Cerebellar deficits were found across diagnostic categories, but especially in those with somatic delusions, perhaps reflecting their older relative age. No correlation with treatment response was noted.

Wolf et al. [27] were specifically interested in delusional infestation, a form of DD in which the person believes, against medical evidence, that he or she is chronically infested with a parasite. Symptoms consist of somatic delusions and tactile hallucinations (sensations of itching and biting). The investigators report on a case series investigating gray- and white-matter brain volumes in 16 such patients and 16 matched healthy controls (mean age 74.1), using structural magnetic resonance imaging. Several regional reductions in gray matter and increases in white matter in patients were found compared to controls. This small study suggests that symptoms in the somatic type of DD have neural correlates, specifically disrupted prefrontal control over somatic sensations. The same group [22], using similar methodology, compared gray-matter volumes in 14 male and 11 female DD patients with nonsomatic paranoid delusions and 25 healthy controls. In this younger-aged series (mean age 53.1; SD 8.1), patients also showed regional aberrations of gray-matter volume as well as abnormal gyrification compared to controls, suggesting that these are possibly correlates of emotions such as fear, anxiety and threat. The authors acknowledge that these conclusions are only speculative and treatment response was not studied. These two studies demonstrate brain-structure abnormalities in DD but do not attempt to correlate them with cognitive changes nor to treatment response.

A one-year study of hospitalized psychiatric patients at least 65 years of age by Fond et al. [25] in France asked whether inappropriately prescribed psychiatric medications (PIP) (that cause cognitive difficulties) were associated with functional outcomes. Data were available for 327 patients; 37.9% were male and the mean age was 73.9. A total of 89 of the patients fell into the category of schizophrenia-related disorders, which includes delusional disorder. Of the full sample, 76% had received what the investigators considered to be inappropriate medications (benzodiazepines and hypnotics). Independently of age, gender and medical/psychiatric diagnosis, the use of PIP was associated, at discharge from hospital, with significantly lower functioning than that in the group who had not received PIP. This suggests that cognition-impairing medications impair functional outcomes, but it is not clear how many of the 89 patients diagnosed with SZ-related disorders suffered from DD.

Harris and collaborators [26] reported on a consecutive case series with the aim of comparing cognitive deficits in late onset DD with those of Alzheimer’s disease (AD). They recruited 19 late-onset DD patients and 20 AD patients from a memory clinic. All participants were comprehensively assessed for executive function, learning and delayed memory, language, processing speed and visuoperceptual skills. Compared with the AD group, the DD patients demonstrated significantly poorer visuoperceptual skills but significantly better memory skills. Because the DD participants in this study were recruited from a memory clinic, this finding may not generalize to all DD patients. Neither sex differences nor treatment response were studied.

Similarly to the previous study, Van Asche et al. [23] set out to clinically and cognitively differentiate functional from neurodegenerative late-onset psychosis. They investigated the symptoms and the neuropsychological profiles of 57 patients with very-late-onset SZ-like psychosis (VLOSLP) (which includes delusional disorder) from patients diagnosed with AD and from those with dementia with Lewy bodies. Members of both latter groups also showed psychotic features. Patients with VLOSLP reported more partition delusions (the belief that people or objects can pass through walls) and auditory hallucinations than the other two groups. The investigators’ conclusion was that despite some differences, the clinical presentation of the three kinds of late-life psychosis and their neuropsychological profiles were not sufficiently distinct to allow an accurate diagnosis to be made without the assistance of imaging techniques. Again, neither sex differences nor treatment responses were studied.

Because case reports speak only of one individual, they cannot be considered as evidence to support or disconfirm a hypothesis. They can, however, be useful in the formation of hypotheses that can then be tested in appropriate trials. For instance, Dua and Grover [21] reported the case of a 75-year-old woman who had suffered from a delusion that she was pregnant for an estimated 19 years. This is an interesting delusion, often attributed to a combination of intense desire for children in infertile women as well as an intense fear in women in whom pregnancy would be socially and economically disastrous [32]. This patient was admitted to hospital for secondary depressive symptoms, which resolved, but the underlying delusion of pregnancy did not respond to a combination of sertraline and olanzapine at standard doses. Magnetic resonance imaging of the brain revealed mild cerebral atrophy with small-vessel ischemic change, and a small right-temporal meningioma. Since the delusion first emerged almost 20 years before the brain changes were found, the association with a lack of treatment response is putative only. This case, however, strongly suggests a clinical trial of brain imaging at the onset of delusions in DD to determine potential associations with structural brain impairment.

D’Auria and collaborators [24] report the case of a 70-year-old woman who had run away from home, her explanation being that she was a burden to her family because of a long-standing parasite infestation. This delusional belief had begun 15 years previously and had occasioned two previous hospitalizations. Psychotherapy plus olanzapine 2.5 mg/daily and sertraline 75 mg/daily were prescribed, with only partial response. Computerized tomography revealed structural brain changes potentially contributory to the delusion and perhaps explaining the relatively poor response to medications. The association of brain changes with lack of response to DD treatment needs a large, methodologically appropriate trial.

Ukai et al. [28] reported the case of a woman in her early 70s with glossodynia diagnosed as DD somatic type because it had lasted for over a decade without improvement and because the patient also suffered persecutory delusions. MRI revealed lacunar infarctions and ischemic changes in thalamus, basal nuclei, pons and deep white matter. There was no response to treatment until the patient was treated with a serotonin-norepinephrine reuptake inhibitor used to treat fibromyalgia (milnacipran 25 mg/day). After a few weeks of treatment, the patient reported disappearance of her oral symptoms and her paranoia. This is an interesting case because of the diagnostic overlap between burning mouth syndrome (BMS) and DD somatic type. BMS is considered to be a pain syndrome of unknown etiology that unpredictably responds to a large variety of therapeutic approaches [33]. This case suggests the hypothesis that some cases of somatic type DD may represent somatic disturbances that lead to secondary paranoid delusions, a hypothesis that may be difficult to investigate. It also shows that even delusions that have lasted over ten years without improvement, and which are associated with substantial brain changes, can improve when the right treatment is found.

In summary, lacunar infarcts, cortical atrophy, alterations in gray- and white-matter volumes as well as cerebellar dysfunctions have been reported. In some studies, they appear to be associated with poor treatment response.

### 3.3. DD in Old Age Is Associated with Self-Harm and Harm to Others (Hypothesis 4)

Three case reports illustrate the potential of self-harm and harm to others in patients with DD in old age.

DD in old age can lead to violence [29]. A 76-year-old man, convinced of his wife’s infidelity, threatened her with physical aggression. His treatment proved difficult because of adverse effects of risperidone. This case underscores the problems of antipsychotic adverse effects in old age as well as the potential dangers to others of delusional jealousy.

A good example of a hypothesis-generating case is the report by Weise et al. [30] of a woman in her mid 60s who presented with attempted suicide in the context of stress related to the COVID-19 pandemic. Her attempt was triggered by complex delusions of persecution imprecisely related to the political controversies surrounding the public response to the pandemic in Germany. This patient refused medication but attained good function attributable to a positive therapeutic relationship and regular outpatient-clinic appointments. The case implies that stress can elicit paranoid delusions and that medication-free psychotherapy can, in such instances, lead to good outcomes. A properly designed trial of these possibilities is warranted. The case also points to the fact that suicide is a chronic risk in this population.

Snowdon [31] describes a 65-year-old woman who believed that her ex-husband was trying to poison her. She responded to treatment with quetiapine. Four years later, however, she was hospitalized with dangerous stab wounds to three areas of her body. She also described taking an overdose a year after her first admission because of continuous harassment by a group that included her ex-husband. She said the harassment had continued and that her house was poisoned. She said “the group” could get to her wherever she went, which led her to feel hopeless. She expressed a fear that her persecutors “might have control over the doctors so I can’t say anything”; “there’s nothing left for me to do but die.” She responded, however, to depot antipsychotics and once-a-month follow ups, and was well at the time of the report.

## 4. Discussion

DD is a nonaffective psychotic disorder with a relatively low prevalence [11,13], first occurring in mid-to-late life. A substantial percentage of patients are first diagnosed in old age, but very little research has been conducted in DD that is specific to the elderly population. In this review, we targeted recent papers on the subject that addressed our initial hypotheses.

We found a small number of studies in DD in older age that examined the separate or combined etiological and treatment-related roles of brain-structure abnormalities, cognitive decline and stressful events. We also came across recent studies that looked at regional differences in prevalence and at the potential role of the prescription of unnecessary drugs and their potentially harmful effects.

With respect to potential risk factors, there are many other acknowledged contributory factors to DD such as genetics [34], immigration [35], minority status [36,37] socioeconomics [38], medical comorbidities [39], sensory impairment [40] and the menopausal loss of estrogen in women [28,41] that have not yet been fully investigated in DD.

The first two hypotheses in our study addressed the incidence, prevalence and sex ratio of DD in old age [16,17]. High rates of DD than usually reported were found, as well as, interestingly, greater incidence in women postmenopause than premenopause. This is potentially attributable to hormonal changes or may be a function of age [17]. The overall prevalence appears to be underestimated in service-based studies as compared with register studies.

The third hypothesis was that poor response to treatment in DD in old age would be associated with brain changes and/or cognitive defects. Several of the studies we reviewed point to the presence of neurobiological correlates of delusions (cerebellar abnormalities [20], alterations in gray and white matter, lacunar infarctions and ischemic brain lesions in DD [19,22,29,30]. Our literature search suggests, but does not prove, that brain lesions can impair the response to psychotropic medications in the elderly age group [20]. To test this, one would need to compare older patients with DD with younger patients showing similar symptom severity.

Duration of delusional symptoms may also be an important dimension of response [42] as the case studies we found demonstrate that patients may not access treatment until many years after symptom onset. Nonadherence to treatment in DD in general, and perhaps particularly in old age, is a vital issue to which insufficient attention has been paid [43,44]. It is also important to note that delusional symptoms in older individuals occur in a wide variety of psychiatric, neuropsychiatric and medical conditions, which are difficult to differentiate from one another [45].

The fourth hypothesis stated that self-harm and aggression is associated with DD in old age. We found three case reports where this was true. Although we did not hypothesize about differential effects of DD subtypes, this issue has been studied in younger DD populations [46]. Somatic delusions may become more frequent with age, as is the case in major depressive disorders [47]. Our cited literature suggests that the jealousy subtype is relatively frequent in older age, an observation that needs better understanding because this form of DD too frequently leads to self-directed and other-directed violence [29].

Our findings show that DD in older age sometimes responds well to either antidepressants and/or antipsychotic medications, but sometimes does not. In this age group, the decision to medicate must involve a careful risk–benefit analysis [45]. This is because these medications induce a variety of serious adverse effects and can lead to premature mortality. On one hand, the study by Nagendra and Snowdon [19] shows substantial sustained improvement from antipsychotic medication. On the other hand, the study by Fond et al. [25] suggests that inappropriate medications are too often prescribed and may lead to reduced function. This finding is supported by Liu et al. [47], who report on potentially serious side effects caused by drug interactions.

Table 2 summarizes conclusions with respect to our four hypotheses.

### Limitations and Strengths

The most important limitation of our review is the scarcity of studies. The small numbers of participants involved in the cited work made it impossible to provide definitive answers to the hypothetical questions we asked. We targeted individuals over age 60, but some studies provided only mean ages, and in many cases, the onset of symptoms had first emerged considerably earlier than the age at ascertainment. It was thus sometimes impossible to determine whether our sample participants could be considered elderly when their symptoms began and whether brain abnormalities and cognitive symptoms found on assessment resulted from age or from DD. Previous work [9] does suggest, however, that cognitive deficits are already present in DD at relatively young ages. Very often, DD patients in our cited studies were included in a heterogeneous diagnostic category, e.g., SZ and related psychoses, making it difficult to be certain whether the results obtained could be applied to the DD subgroup. These limitations in the available literature have been signaled previously, as noted in a Cochrane review [48]. The main strength of our review is to raise important questions and highlight gaps in research that need to be filled.

## 5. Conclusions

Data on delusional disorder (DD) in elderly populations are limited because the prevalence of DD is relatively low to start with and cases are often not brought to medical attention. There is also considerable overlap between the symptoms of DD and other psychoses such as schizophrenia, affective disorders with psychotic features and neurologic diseases with psychotic features, which adds to the difficulty of ascertainment. Methodological shortcomings of the studies we found undermined their ability to definitively confirm or disconfirm our hypotheses about prevalence, gender aspects, treatment response, self-harm and risk of harm to others in old-age DD.

With regard to the first and second hypotheses (epidemiological, gender-specific), there are indications that DD is more prevalent in old age than it is in earlier years. The data on male/female ratios are unclear, but it is interesting, and perhaps significant, to note that in women, DD tends to begin after menopause, suggesting a neuroprotective effect of estrogens that is lost after that time.

Brain changes are definitely demonstrable but nonspecific, and their relationship with cognitive defects and poor pharmacological response is unconvincing (hypothesis 3). Future studies should specifically investigate the correlation between cognitive defects and/or brain structures and response to treatment. The results may be easier to interpret in younger populations because age itself is a confounding factor.

The risk for aggression and suicide is evident in the case reports but its prevalence cannot be substantiated (hypothesis 4). Again, old age, loss of resources and support and declining general health is a potential risk factor for suicidal behavior.

Psychological interventions, in the context of elderly DD, are underutilized. They may be a safe option that has been proven to be effective as an adjunctive treatment in patients with persistent delusions. Testing the effectiveness of specific forms of psychotherapy in aging patients with DD promises to be a fruitful field of future research.

What this review was able to do was to shine a spotlight on the necessary direction of future clinical trials. Investment needs to be made in well-controlled longitudinal studies that test specific hypotheses and that include large, representative and randomized samples.

## Figures and Tables

**Table 1:** Main characteristics of studies addressing epidemiology, risk factors, clinical features and treatment outcomes in elderly DD patients (n = 16).

Authors and Year of Publication	Study Design	Method	Women (%), Age [Mean, (SD)]	Hypothesis Addressed
Hypotheses 1 and 2				
**Castro-de-Araujo et al., 2020** [16]	Register study of mental-illness prevalence by diagnosis in the elderly	Outpatient visit rates of seniors over age 60 throughout Brazil	F = 340.250 (30.3%)	Suggests, but does not prove, higher-than-expected **prevalence** of DD in Brazilian seniors. No male/female differences are mentioned
**González-Rodríguez et al., 2015** [17]	Prospective observational study of women with DD, to determine the effect of pre- and postmenopausal onset	Consecutive cases of women assessed at baseline and after 24 months.	80 (100%) 57 completed 2-year follow up. Premenopausal onset = 25 Postmenopausal onset + 55	**Women’s** onset is more often postmenopausal, with symptom differences between pre- and postmenopausal onset
**Meesters et al., 2012** [18]	Case register study to determine 1 year prevalence, onset ages and sex ratios of patients with psychosis aged 60 and over	Search of the computerized mental health records over 1 year in Amsterdam	DD: n = 8 (4.4%)	1 year **prevalence** of DD in old age was 0.03% and in **women**, was found only in those with onset over age 40. Very small numbers.
**Hypothesis 3**				
**Nagendra and Snowdon, 2020** [19]	Retrospective 12-year study of 55 DD patients over age 65 to study the effect of treatment	Follow up after a mean duration of 36.6 months	F = 39 (71%) Age = 74.5 (7.65)	Treatment response was 55%. Six patients developed **cognitive** impairments at follow up, but no association with treatment response was mentioned.
**Krämer et al., 2020** [20]	Cross-sectional study designed to study cerebellar dysfunction in psychosis, including somatic and nonsomatic DD	Structural MRI in DD somatic type, DD nonsomatic type, schizophrenia and healthy controls	DD somatic type: 8 (6 women) mean age: 72.6 (9.3) Nonsomatic DD: 13 females mean age: 55.9	Substantial **cerebellar impairment**, particularly in DD somatic type, related to age but no mention of treatment response
**Dua and Grover, 2020** [21]	Case report of DD of long duration, with MRI brain changes	Woman with long-term delusion of pregnancy and resistance to treatment	75-year-old woman with DD (delusions of pregnancy ICD-10) for 19 years	**MRI brain changes** potentially associated with lack of **treatment response**
**Wolf et al., 2020** [22]	Case series of patients with DD paranoid type in search of brain correlates	Brain structural neuroimaging study of patients and matched healthy controls	14 patients Sex unspecified	Aberrant **gray-matter volume** in right prefrontal regions in paranoid type-DD.
**Van Asche et al., 2019** [23]	Case register study to differentiate 3 groups of late-life psychoses on the basis of symptoms, neuropsychological profile	Comparison of symptoms and neuropsychological profiles of functional and organic psychoses	n= 57 in very-late-onset SZ-like group. 77.8% were women. Mean age: 79.25	Differences found in symptoms but not in **cognitive** profiles. Neither treatment responses nor sex differences were studied
**D’Auria et al., 2018** [24]	Case report of DD lasting 15 years with cortical atrophy and nonresponse to Rx	Woman with DD nonresponsive to psychotherapy and pharmacotherapy	70-year-old woman with a 15-year history of delusional parasitosis.	CT: microvascular ischemic **cortical changes** and **nonresponse**.
**Fond et al., 2016** [25]	Retrospective 1-year study of cognition-impairing medications on function of patients aged 65 and over	Chart review of function in elderly psychiatric patients at hospital discharge	Total: n = 327 F = 62% Mean age: 73.9	**Cognition**-impairing medications may reduce function. Precise diagnosis was unclear in this study
**Harris et al., 2014** [26]	Case series comparing cognitive deficits fin late-onset DD with those in Alzheimer’s disease	Memory clinic cases (19 DD, 20 AD) assessed by a comprehensive neuropsychological battery	Total DD: n = 19 Sex not specified	Significant **cognitive** impairment found in late onset DD, but correlation with treatment response was not attempted
**Wolf et al., 2013** [27]	Case series of patients with somatic DD to determine presence of neural correlates	Brain structural neuroimaging study of patients and matched controls	16 patients with delusion of parasitosis; 9 were women. Mean age: 74.1	**Gray- and white-matter** volume differences were found. Rx response not studied
**Ukai et al., 2013** [28]	Case report of effective treatment of long-term somatic-type DD with brain changes	10-year history of DD somatic type (orofacial region) who, after many different treatment trials, responded.	Age: early 70s	**Brain changes** on MRI. Good **response** after 10 years due to milnacipran (25 mg/day)
**Hypothesis 4**				
**Machado et al., 2021** [29]	Case report of male DD in old age and the threat of violence	Case of DD jealous type, with potential aggression, and adverse effects of antipsychotics	76-year-old man	DD can lead to **physical aggression**. The case also illustrates adverse effects of antipsychotics.
**Weise et al., 2020** [30]	Case report of stress-induced delusions, a suicide attempt and medication-free Rx	Woman with complex paranoid delusions and a suicide attempt triggered by stress	Age: mid-sixties woman with persecutory delusions (ICD-10)	Points to the threat of **suicide** in DD
**Snowdon, 2017** [31]	Case report: Woman with DD, hyperthyroidism and multiple suicide attempts	DD persecutory type, suicide attempts, eventual good response to treatment	Age: 65	Points out **suicide** risk in DD

**Table 2:** Tentative conclusions.

**(1) Incidence and prevalence of DD in old age**	
	Prevalence of DD is underestimated in service-based studies. Higher rates of DD in this group are found in register studies.
**(2) Gender aspects of DD in old age**	
	Cases in women tend to begin after menopause.
**(3) DD in old age is associated with brain changes and cognitive defects that may impair treatment**	
	Microvascular ischemic findings (lacunar infarcts), cortical atrophy, alterations in gray- and white-matter volumes, and cerebellar dysfunctions have been reported, sometimes associated with poor treatment response.
**(4) Self-harm and aggression in DD in old age**	
	Prevention of suicide and violence in DD requires targeted intervention.

## Data Availability

The data presented in this review are available upon request from the corresponding author.
